# Crystal structure of tetra­iso­butyl­thiuram di­sulfide

**DOI:** 10.1107/S2056989017015158

**Published:** 2017-10-24

**Authors:** Patricia R. Fontenot, Bo Wang, Yueli Chen, James P. Donahue

**Affiliations:** a Department of Chemistry, Tulane University, 6400 Freret Street, New Orleans, Louisiana 70118-5698, USA; b Department of Chemistry, SUNY Stony Brook, 100 Nicolls Road, Stony Brook, New York 11790-3400, USA

**Keywords:** crystal structure, tetra­thiuram di­sulfide, di­thio­carbamate, precursor, weak S⋯H—C inter­action

## Abstract

Tetra­kis(2-methyl­prop­yl)thio­per­oxy­dicarbonic di­amide, or tetra­iso­butyl­thiuram di­sulfide, C_18_H_36_N_2_S_4_, crystallizes in a general position in the triclinic space group *P*-1 but shows pseudo-*C*_2_ symmetry about the di­sulfide bond. The C—S—S—C torsion angle [−85.81 (2)°] and the dihedral angle between the two NCS_2_ mean planes [85.91 (5)°] are within the range observed for this compound type. Multiple intra- and inter­molecular S⋯H—C close contacts appear to play a role in assisting the specific conformation of the pendant isobutyl groups and the packing arrangement of mol­ecules within the cell. Tetra­iso­butyl­thiuram di­sulfide mol­ecules of one optical configuration form sheets in the plane of the *a* and *b* axes. Inversion centers exist between adjoining sheets, which stack along the *c* axis and alternate in the handedness of their constituent mol­ecules.

## Abstract 2

The crystal structure of tetra­iso­butyl­thiuram di­sulfide reveals a −85.81 (1)° C—S—C—S torsion angle and multiple intra- and inter­molecular S⋯C—H close contacts.

## Chemical context   

*N*,*N*,*N*′,*N*′-Tetra­alkyl­thio­per­oxy­dicarbonic di­amides, com­mon­ly called tetra­thiuram di­sulfides, comprise a class of organosulfur compounds with applications that are both diverse and long-standing. Tetra­methyl­thiuram di­sulfide, known by the commercial name thiram, is broadly useful both as a fungicide (Sharma *et al.*, 2003 ▸) and as a repellent against animals that feed upon seedling trees (Radwan, 1969 ▸). In industry, thiram and related tetra­alkyl­thiuram di­sulfides find application as vulcanizing agents in the production of synthetic rubber (Datta & Ingham, 2001 ▸; Ignatz-Hoover & To, 2016 ▸). Tetra­ethyl­thiuram di­sulfide, under the trade name disulfiram, is used for the treatment of chronic alcoholism because of its inhibitory effect upon liver alcohol de­hydrogenase (Mutschler *et al.*, 2016 ▸). More recently, it has received scrutiny for its ability to sensitize cancer cells to radiotherapy and to the effects of anti­cancer drugs (Jiao *et al.*, 2016 ▸) as well as for its bactericidal action against drug-resistant *Mycobacterium tuberculosis* (Horita *et al.*, 2012 ▸). Tetra­alkyl­thiuram di­sulfides function both as chelating ligands themselves (Chieh, 1977 ▸; Chieh, 1978 ▸; Thirumaran *et al.*, 2000 ▸; Saravanan *et al.*, 2005 ▸; Prakasam *et al.*, 2009 ▸) and as precursors to di­thio­carbamate ligands, which are used in the coordination chemistry of both the transition metals (Hogarth, 2005 ▸) and main group elements (Heard, 2005 ▸).

In the course of some studies of diiso­butyl­dithio­carbamate coordination complexes of molybdenum, we have noted a report describing an ^1^H NMR spectrum of [Ni(S_2_CN^*i*^Bu_2_)_2_] that was more complex than anti­cipated, even considering the hindered rotation about the ^−^S_2_–CN^*i*^Bu_2_ bond (Raston & White, 1976 ▸). This complexity was attributed to intra­ligand S⋯H inter­actions involving the tertiary hydrogen of the isobutyl group. Although the room temperature ^1^H NMR spectrum of *N*,*N*,*N*′,*N*′- tetra­kis­(2-methyl­prop­yl)thio­per­oxy­dicarbonic di­amide (tetra­iso­butyl­thiuram di­sulfide) itself does not show evidence of such intra­molecular inter­action, several recent studies of tetra­thiuram di­sulfides have suggested such inter­actions in the crystalline state (Raya *et al.*, 2005 ▸; Srinivasan *et al.*, 2012 ▸; Nath *et al.*, 2016 ▸). This possibility of similar weak inter­action(s) in the crystal structure of tetra­iso­butyl­thiuram di­sulfide has motivated a determination of its structure by X-ray diffraction, reported herein.
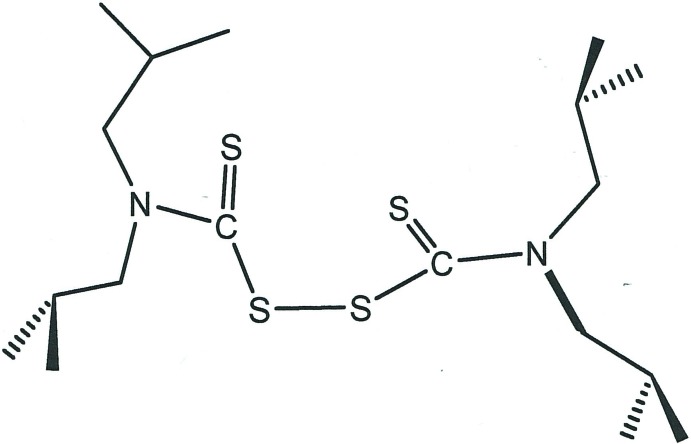


## Structural commentary   

**Figure 1:**
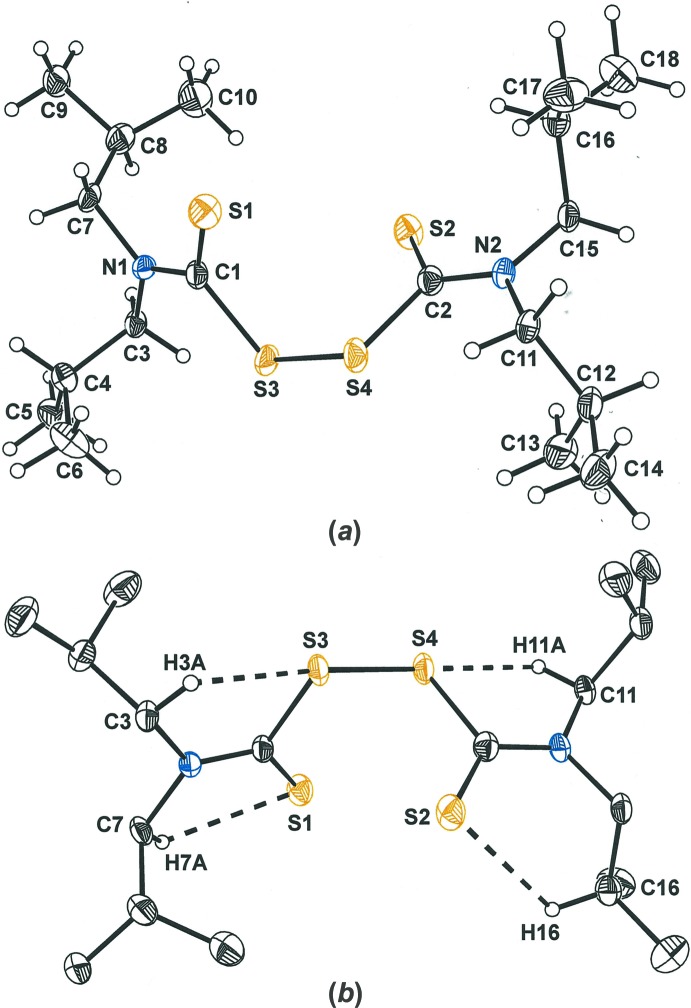
(*a*) Displacement ellipsoid plot (50%) of tetra­iso­butyl­thiuram di­sulfide with complete labeling for the non-H atoms. (*b*) Displacement ellipsoid plot (50% probability) of tetra­iso­butyl­thiuram di­sulfide illustrating close intra­molecular S⋯H—C contacts (dashed lines).

Tetra­iso­butyl­thiuram di­sulfide crystallizes upon a general position in *P* 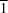 but has pseudo-*C*_2_ symmetry across the di­sulfide bond, strict *C*_2_ symmetry being disrupted by conformational differences among the pendant isobutyl groups (Fig. 1 ▸ *a*). Despite the lack of strict *C*_2_ symmetry, tetra­iso­butyl­thiuram di­sulfide is nevertheless chiral. The image in Fig. 1 ▸ *a* presents the mol­ecule with a left-handed configuration to the core –H_2_CNC(S)S–SC(S)NCH_2_– portion. If Fig. 1 ▸ *a* were to be viewed from above, along the pseudo *C*_2_ axis that bis­ects the S3—S4 bond, the C1—S1 and C2—S2 thione bonds would project forward and backward, respectively, from the plane of the paper and thereby define a left-handed propeller. The right-handed counterpart is necessarily the other occupant of the unit cell, as required by the racemic space group. Among the structurally characterized thiuram di­sulfides, crystallographically imposed *C*_2_ symmetry is also common (Fig. 3 ▸).

The S3—S4 bond length is 1.9931 (10) Å, while the thione C=S bonds are essentially identical at 1.642 (3) and 1.643 (3) Å. The C1—S3—S4—C2 torsion angle, τ, is −85.81 (2)° and, as is typical of tetra­thiuram di­sulfides, very similar in magnitude to the angle of 85.91 (5)° between the mean planes defined by the S_2_CN fragments, θ.

**Figure 2:**
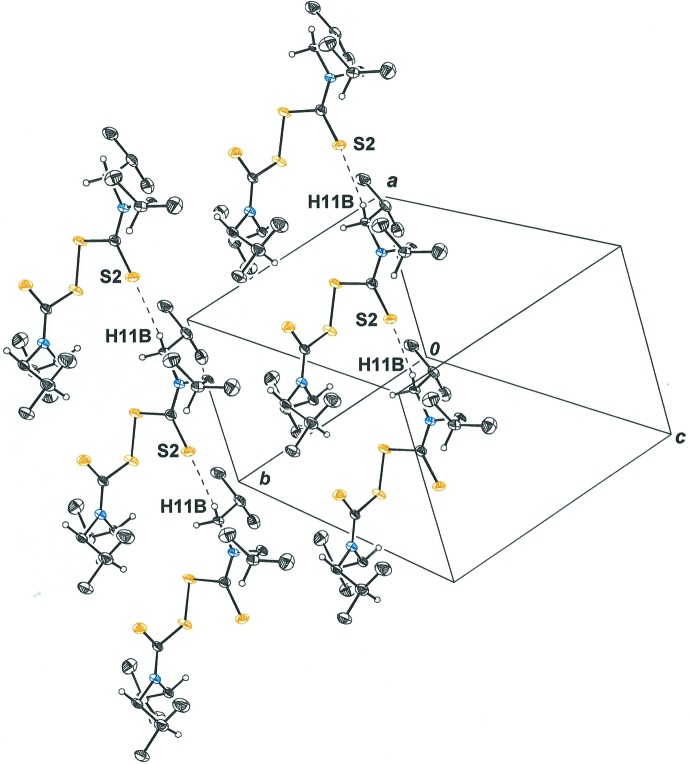
Stacking of tetra­iso­butyl­thiuram di­sulfide mol­ecules along the *a* axis of the unit cell, showing inter­molecular S⋯H—C close contacts. Displacement ellipsoids are represented at the 50% probability level. Parallel stacks fill in the *ab* plane to form two-dimensional sheets, as shown. (Symmetry operations: *x* + 1, *y*, *z*; *x*, *y* + 1, *z*.)

Multiple intra­molecular S⋯H–C contacts that are shorter than, or close to, the 2.92 Å sum of the van der Waals radii (Rowland & Taylor, 1996 ▸) for sulfur and hydrogen are calculated for the structure of tetra­iso­butyl­thiuram di­sulfide. Each of the four sulfur atoms on the mol­ecule is a participant in such a close contact, as illustrated in Fig. 1 ▸ *b* and shown in Table 1 ▸. Although weak individually, particularly since these *D*—H⋯*A* angles are closer to 90° than to 180° (Table 1 ▸), these inter­actions may act cooperatively with packing forces to decide the specific mol­ecular conformation that is adopted. Weak inter­molecular S⋯H—C contacts are also calculated for mol­ecules that stack along the *a* axis of the cell (Fig. 2 ▸). While angles for these contacts are larger (145.6, 159.5°), the *D*⋯*A* separations are longer [3.834 (3), 3.810 (3) Å]. The geometric parameters for both these intra­molecular and inter­molecular S⋯C–H contacts fall within the range defined as consistent with a weak *D*—H⋯A inter­action (Desiraju & Steiner, 1999 ▸). These features of the mol­ecular packing in the crystal structure of tetra­iso­butyl­thiuram di­sulfide suggest that the crystal structures of coordination complexes with the diiso­butyl­dithio­carbamate ligand be considered for similar S⋯H—C contacts and, importantly, that variable temperature ^1^H NMR spectroscopy be used to assess the importance of any such inter­actions in solution.

## Supra­molecular features   

Mol­ecules of tetra­iso­butyl­thiuram di­sulfide are linked by C—H⋯S hydrogen bonds (Table 1 ▸) to form linear chains directed along the *a* axis of the cell, and parallel chains then align within the *ab* plane to form sheets (Fig. 2 ▸). Because the mol­ecules within a single sheet are related, one from another, only by translations along *a* or *b*, they all have the same optical configuration. The sheets in the *ab* plane then stack along the *c* axis of the cell. The cell’s inversion center resides within the center of the cell and relates mol­ecules from neighboring sheets. Consequently, the sheets alternate in the handedness of the mol­ecules from which they are comprised.

## Database survey   

Values for τ and θ for structures in the Cambridge Structural Database (Web CSD v1.1.1; Groom *et al.*, 2016 ▸) were determined using Mercury (Macrae *et al.*, 2008 ▸). These structures are: METHUS (Marøy, 1965 ▸), METHUS01 (Ymén, 1983 ▸), METHUS02 (Wang *et al.*, 1986 ▸), METHUS03 (Wang & Liao, 1989 ▸), METHUS04 (Wang & Liao, 1989 ▸), ETHUSS (Karle *et al.*, 1967 ▸) ETHUSS01 (Wang *et al.*, 1986 ▸), ETHUSS02 (Wang & Liao, 1989 ▸), ETHUSS03 (Wang & Liao, 1989 ▸), ETHUSS04 (Shi & Wang, 1992 ▸), ETHUSS05 (Hu, 2000 ▸), HIQJUM (Jian *et al.*, 1999 ▸), HIQJUM01 (Yu & Wang, 2003 ▸), JECYAZ (Kumar *et al.*, 1990 ▸), TIBFEQ (Zhai *et al.*, 2007 ▸), ZEMPUC (Hall & Tiekink, 1995 ▸), KAZHEA (Karim *et al.*, 2012 ▸), NELTUT (Fun *et al.*, 2001 ▸), XEBJOF (Ajibade *et al.*, 2012 ▸), JAXPOO (Raya *et al.*, 2005 ▸), CAPLEK (Williams *et al.*, 1983 ▸), CAPLEK01 (Ymén, 1983 ▸), CAPLEK02 (Yamin *et al.*, 1996 ▸), CAPLEK03 (Bai *et al.*, 2010 ▸), RISNEN (Quan *et al.*, 2008 ▸), ULOXIC (Bodige & Watson, 2003 ▸), PIPTHS (Dix & Rae, 1973 ▸), PIPTHS01 (Shi & Wang, 1992 ▸), EWESUW (Nath *et al.*, 2016 ▸), BOMPAU (Rout *et al.*, 1982 ▸), VOHFIH (Polyakova & Starikova, 1990 ▸), VOHFIH01 (Ivanov *et al.*, 2003 ▸), PECWOL (Uludağ *et al.*, 2013 ▸), ZIJLOV (Srinivasan *et al.*, 2012 ▸) and MEMFUG (Sączewski *et al.*, 2006 ▸).

**Figure 3:**
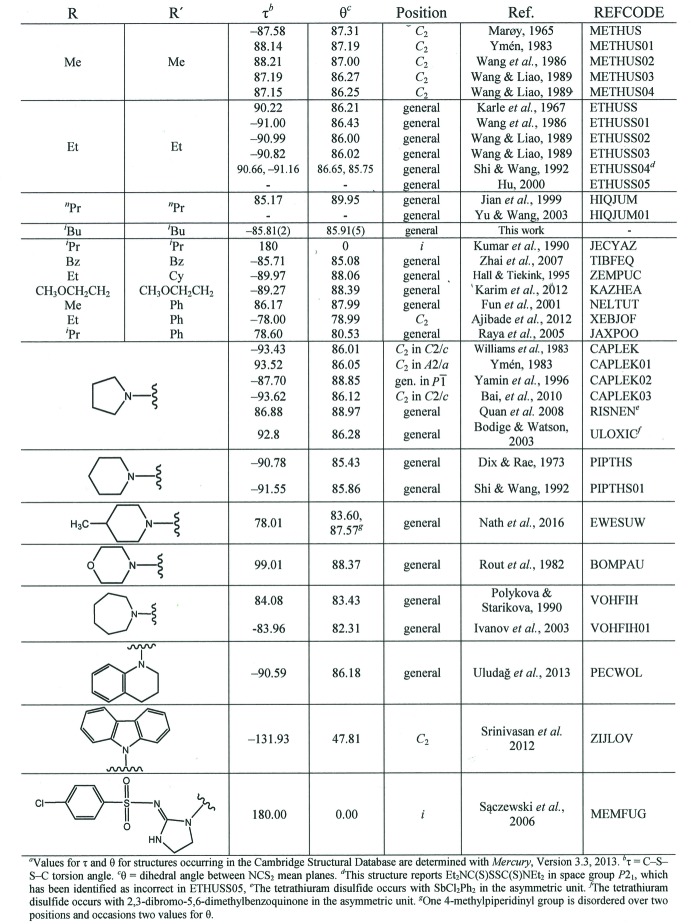
Summary of structurally characterized tetra­thiuram di­sulfides, *RR*’NC(S)SSC(S)N*RR*’.

The C—S—S—C torsion angle (τ) and the dihedral angle (θ) between S_2_CN mean planes are closely comparable to values observed for the analogous features in most other tetra­thiuram di­sulfides, as summarized in Fig. 3 ▸. Positive and negative values of τ occur with approximately equal frequency for tetra­thium di­sulfides that have been characterized structurally by X-ray diffraction (Fig. 3 ▸). For those which do not reside on an inversion center (Kumar *et al.*, 1990 ▸; Sączewski *et al.*, 2006 ▸) or have conformations obviously perturbed by inter­molecular inter­actions involving the pendant groups on nitro­gen (Srinivasan *et al.*, 2012 ▸), the average of the absolute value of τ is 88.4°, and the range is 78.0–99.0°. Similarly, the average value of θ is 86.1°, with a range of 79.0–90.0°.

## Synthesis and crystallization   

The synthesis procedure employed was that described by Kapanda *et al.*, 2009 ▸. Pale-yellow block-shaped crystals of tetra­iso­butyl­thiuram di­sulfide (m.p. 343 K) were obtained by slow evaporation of a CH_2_Cl_2_ solution. ^1^H NMR (δ, ppm in DMSO-*d*_6_): 3.83 [*d*, *J* = 12 Hz, 8H, –C*H*_2_CH(CH_3_)_2_], 2.39 [*br m*, 4H, –CH_2_C*H*(CH_3_)_2_], 0.98 [*d*, *J* = 8 Hz, 12H, –CH_2_CH(C*H*_3_)_2_], 0.87 [*d*, *J* = 8 Hz, 12H, –CH_2_CH(C*H*_3_)_2_].

## Refinement details   

Crystal data, data collection and structure refinement details are summarized in Table 2 ▸. Hydrogen atoms were added in calculated positions and refined with isotropic displacement parameters that were approximately 1.2 times (for –CH– and –CH_2_) or 1.5 times (for –CH_3_) those of the carbon atoms to which they were attached. The C—H distances assumed were 1.00, 0.99, and 0.98 Å for the –CH–, –CH_2_, and –CH_3_ types of hydrogen atoms, respectively.

## Supplementary Material

Crystal structure: contains datablock(s) I, global. DOI: 10.1107/S2056989017015158/lh5851sup1.cif

Structure factors: contains datablock(s) I. DOI: 10.1107/S2056989017015158/lh5851Isup2.hkl

Supporting information file. DOI: 10.1107/S2056989017015158/lh5851Isup3.cml

CCDC reference: 1580550

Additional supporting information:  crystallographic information; 3D view; checkCIF report

## Figures and Tables

**Table 1:** Hydrogen-bond geometry (Å, °)

*D*—H⋯*A*	*D*—H	H⋯*A*	*D*⋯*A*	*D*—H⋯*A*
C3—H3*A*⋯S3	0.99	2.34	2.907 (3)	115
C7—H7*A*⋯S1	0.99	2.60	3.084 (3)	110
C8—H8⋯S1^i^	1.00	2.97	3.834 (3)	146
C11—H11*A*⋯S4	0.99	2.33	2.896 (3)	115
C11—H11*B*⋯S2^ii^	0.99	2.87	3.810 (3)	160
C16—H16⋯S2	1.00	2.91	3.473 (3)	117

Symmetry codes: (i) 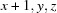; (ii) 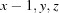.

**Table 2:** Experimental details

Crystal data	
Chemical formula	C_18_H_36_N_2_S_4_
*M*_r_	408.73
Crystal system, space group	Triclinic, *P* 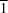
Temperature (K)	100
*a*, *b*, *c* (Å)	7.2449 (11), 9.6102 (14), 17.196 (3)
α, β, γ (°)	98.580 (2), 94.540 (2), 103.409 (2)
*V* (Å^3^)	1143.5 (3)
*Z*	2
Radiation type	Mo *K*α
μ (mm^−1^)	0.42
Crystal size (mm)	0.17 × 0.12 × 0.06

Data collection	
Diffractometer	Bruker APEXII CCD
Absorption correction	Multi-scan (*SADABS*; Bruker, 2016 ▸)
*T*_min_, *T*_max_	0.745, 0.977
No. of measured, independent and observed [*I* > 2σ(*I*)] reflections	17180, 4168, 3161
*R*_int_	0.057
(sin θ/λ)_max_ (Å^−1^)	0.604

Refinement	
*R*[*F* ^2^ > 2σ(*F* ^2^)], *wR*(*F* ^2^), *S*	0.052, 0.146, 1.07
No. of reflections	4168
No. of parameters	225
H-atom treatment	H-atom parameters constrained
Δρ_max_, Δρ_min_ (e Å^−3^)	0.87, −0.35

Computer programs: *APEX3* and *SAINT* (Bruker, 2016 ▸), *SHELXT* (Sheldrick, 2015*a* ▸), *SHELXL2014* (Sheldrick, 2015*b* ▸) and *SHELXTL* (Sheldrick, 2008 ▸).

## supplementary crystallographic information

### Crystal data

**Table:**

C_18_H_36_N_2_S_4_	*Z* = 2
*M**_r_* = 408.73	*F*(000) = 444
Triclinic, *P*1	*D*_x_ = 1.187 Mg m^−^^3^
*a* = 7.2449 (11) Å	Mo *K*α radiation, λ = 0.71073 Å
*b* = 9.6102 (14) Å	Cell parameters from 4848 reflections
*c* = 17.196 (3) Å	θ = 2.2–25.2°
α = 98.580 (2)°	µ = 0.42 mm^−^^1^
β = 94.540 (2)°	*T* = 100 K
γ = 103.409 (2)°	Block, pale yellow
*V* = 1143.5 (3) Å^3^	0.17 × 0.12 × 0.06 mm

### Data collection

**Table:**

Bruker APEXII CCD diffractometer	4168 independent reflections
Radiation source: fine-focus sealed tube	3161 reflections with *I* > 2σ(*I*)
Graphite monochromator	*R*_int_ = 0.057
Detector resolution: 8.3333 pixels mm^-1^	θ_max_ = 25.4°, θ_min_ = 2.2°
φ and ω scans	*h* = −8→8
Absorption correction: multi-scan (*SADABS*; Bruker, 2016)	*k* = −11→11
*T*_min_ = 0.745, *T*_max_ = 0.977	*l* = −20→20
17180 measured reflections	

### Refinement

**Table:**

Refinement on *F*^2^	Primary atom site location: structure-invariant direct methods
Least-squares matrix: full	Hydrogen site location: inferred from neighbouring sites
*R*[*F*^2^ > 2σ(*F*^2^)] = 0.052	H-atom parameters constrained
*wR*(*F*^2^) = 0.146	*w* = 1/[σ^2^(*F*_o_^2^) + (0.0883*P*)^2^] where *P* = (*F*_o_^2^ + 2*F*_c_^2^)/3
*S* = 1.07	(Δ/σ)_max_ = 0.001
4168 reflections	Δρ_max_ = 0.87 e Å^−^^3^
225 parameters	Δρ_min_ = −0.35 e Å^−^^3^
0 restraints	

### Special details

**Table:**

Experimental. The diffraction data were obtained from 3 sets of 400 frames, each of width 0.5° in ω, collected at φ = 0.00, 90.00 and 180.00° and 2 sets of 800 frames, each of width 0.45° in φ, collected at ω = –30.00 and 210.00°. The scan time was 60 sec/frame.
Geometry. All esds (except the esd in the dihedral angle between two l.s. planes) are estimated using the full covariance matrix. The cell esds are taken into account individually in the estimation of esds in distances, angles and torsion angles; correlations between esds in cell parameters are only used when they are defined by crystal symmetry. An approximate (isotropic) treatment of cell esds is used for estimating esds involving l.s. planes.

### Fractional atomic coordinates and isotropic or equivalent isotropic displacement parameters (Å^2^)

**Table:**

	*x*	*y*	*z*	*U*_iso_*/*U*_eq_	
S1	0.11097 (10)	0.22088 (8)	0.72835 (4)	0.0265 (2)	
S2	0.35046 (10)	0.62092 (8)	0.68215 (4)	0.0268 (2)	
S3	0.37169 (10)	0.47724 (7)	0.84025 (4)	0.0250 (2)	
S4	0.13548 (10)	0.54581 (8)	0.82084 (4)	0.0253 (2)	
N1	0.4760 (3)	0.2440 (2)	0.77920 (12)	0.0177 (5)	
N2	0.0291 (3)	0.6939 (2)	0.71490 (12)	0.0184 (5)	
C1	0.3217 (4)	0.2980 (3)	0.77839 (15)	0.0195 (6)	
C2	0.1689 (4)	0.6296 (3)	0.73258 (16)	0.0208 (6)	
C3	0.6574 (4)	0.3090 (3)	0.83096 (15)	0.0195 (6)	
H3A	0.6641	0.4125	0.8506	0.023*	
H3B	0.7650	0.3052	0.7993	0.023*	
C4	0.6811 (4)	0.2335 (3)	0.90154 (15)	0.0253 (6)	
H4	0.6567	0.1270	0.8813	0.030*	
C5	0.8874 (4)	0.2875 (4)	0.94048 (18)	0.0375 (8)	
H5A	0.9155	0.3924	0.9597	0.056*	
H5B	0.9740	0.2674	0.9017	0.056*	
H5C	0.9052	0.2375	0.9851	0.056*	
C6	0.5418 (5)	0.2552 (4)	0.96030 (17)	0.0384 (8)	
H6A	0.5722	0.3576	0.9854	0.058*	
H6B	0.5511	0.1948	1.0009	0.058*	
H6C	0.4115	0.2273	0.9328	0.058*	
C7	0.4768 (4)	0.1089 (3)	0.72601 (15)	0.0215 (6)	
H7A	0.3433	0.0528	0.7085	0.026*	
H7B	0.5411	0.0491	0.7556	0.026*	
C8	0.5774 (4)	0.1370 (3)	0.65392 (16)	0.0295 (7)	
H8	0.7063	0.2031	0.6734	0.035*	
C9	0.6088 (4)	−0.0045 (3)	0.61130 (17)	0.0303 (7)	
H9A	0.6814	−0.0465	0.6480	0.045*	
H9B	0.6803	0.0145	0.5664	0.045*	
H9C	0.4849	−0.0727	0.5920	0.045*	
C10	0.4765 (5)	0.2120 (4)	0.59941 (19)	0.0437 (9)	
H10A	0.5528	0.2336	0.5562	0.066*	
H10B	0.4598	0.3026	0.6291	0.066*	
H10C	0.3510	0.1484	0.5775	0.066*	
C11	−0.1242 (4)	0.7070 (3)	0.76450 (15)	0.0191 (6)	
H11A	−0.1361	0.6320	0.7988	0.023*	
H11B	−0.2462	0.6864	0.7296	0.023*	
C12	−0.0943 (4)	0.8561 (3)	0.81718 (16)	0.0266 (7)	
H12	−0.1195	0.9262	0.7826	0.032*	
C13	0.1058 (4)	0.9157 (3)	0.86038 (18)	0.0337 (7)	
H13A	0.1164	1.0126	0.8906	0.051*	
H13B	0.1988	0.9220	0.8218	0.051*	
H13C	0.1316	0.8510	0.8966	0.051*	
C14	−0.2443 (5)	0.8413 (3)	0.87450 (18)	0.0353 (7)	
H14A	−0.2247	0.7710	0.9081	0.053*	
H14B	−0.3719	0.8077	0.8446	0.053*	
H14C	−0.2329	0.9358	0.9077	0.053*	
C15	0.0346 (4)	0.7671 (3)	0.64587 (15)	0.0209 (6)	
H15A	0.1683	0.8198	0.6436	0.025*	
H15B	−0.0420	0.8400	0.6531	0.025*	
C16	−0.0405 (4)	0.6652 (3)	0.56672 (15)	0.0232 (6)	
H16	0.0321	0.5883	0.5614	0.028*	
C17	−0.2504 (4)	0.5917 (4)	0.56081 (18)	0.0360 (8)	
H17A	−0.3243	0.6653	0.5659	0.054*	
H17B	−0.2725	0.5343	0.6033	0.054*	
H17C	−0.2908	0.5276	0.5094	0.054*	
C18	0.0007 (5)	0.7523 (3)	0.50071 (17)	0.0385 (8)	
H18A	−0.0641	0.8315	0.5064	0.058*	
H18B	−0.0459	0.6888	0.4494	0.058*	
H18C	0.1388	0.7927	0.5036	0.058*	

### Atomic displacement parameters (Å^2^)

**Table:**

	*U*^11^	*U*^22^	*U*^33^	*U*^12^	*U*^13^	*U*^23^
S1	0.0192 (4)	0.0294 (4)	0.0315 (4)	0.0091 (3)	0.0001 (3)	0.0041 (3)
S2	0.0186 (4)	0.0343 (4)	0.0328 (4)	0.0133 (3)	0.0059 (3)	0.0101 (3)
S3	0.0285 (4)	0.0215 (4)	0.0278 (4)	0.0155 (3)	−0.0030 (3)	0.0013 (3)
S4	0.0285 (4)	0.0274 (4)	0.0283 (4)	0.0188 (3)	0.0074 (3)	0.0100 (3)
N1	0.0191 (12)	0.0171 (11)	0.0176 (11)	0.0076 (9)	−0.0006 (9)	0.0013 (9)
N2	0.0163 (11)	0.0180 (11)	0.0235 (12)	0.0087 (9)	0.0031 (9)	0.0045 (9)
C1	0.0240 (15)	0.0182 (13)	0.0206 (14)	0.0106 (11)	0.0053 (11)	0.0070 (11)
C2	0.0195 (14)	0.0196 (14)	0.0237 (14)	0.0061 (11)	0.0002 (11)	0.0037 (11)
C3	0.0184 (14)	0.0165 (13)	0.0243 (14)	0.0066 (11)	−0.0010 (11)	0.0039 (11)
C4	0.0280 (16)	0.0263 (15)	0.0239 (15)	0.0103 (12)	−0.0007 (12)	0.0075 (12)
C5	0.0311 (18)	0.053 (2)	0.0319 (17)	0.0170 (15)	−0.0057 (13)	0.0111 (15)
C6	0.0363 (18)	0.054 (2)	0.0283 (17)	0.0109 (16)	0.0034 (14)	0.0167 (15)
C7	0.0260 (15)	0.0158 (13)	0.0255 (15)	0.0111 (11)	0.0052 (12)	0.0020 (11)
C8	0.0365 (18)	0.0273 (16)	0.0288 (16)	0.0152 (14)	0.0092 (13)	0.0033 (13)
C9	0.0390 (18)	0.0301 (16)	0.0289 (16)	0.0204 (14)	0.0079 (13)	0.0068 (13)
C10	0.059 (2)	0.048 (2)	0.0371 (19)	0.0299 (18)	0.0191 (17)	0.0126 (16)
C11	0.0152 (13)	0.0184 (13)	0.0267 (14)	0.0088 (11)	0.0041 (11)	0.0047 (11)
C12	0.0342 (17)	0.0212 (14)	0.0301 (16)	0.0143 (13)	0.0071 (13)	0.0088 (12)
C13	0.0413 (19)	0.0250 (16)	0.0339 (17)	0.0071 (14)	0.0042 (14)	0.0040 (13)
C14	0.0410 (19)	0.0326 (17)	0.0377 (18)	0.0196 (15)	0.0118 (15)	0.0033 (14)
C15	0.0216 (14)	0.0179 (13)	0.0258 (15)	0.0101 (11)	0.0001 (11)	0.0050 (11)
C16	0.0206 (14)	0.0256 (14)	0.0257 (15)	0.0102 (12)	0.0020 (11)	0.0048 (12)
C17	0.0275 (17)	0.0418 (19)	0.0328 (17)	0.0045 (14)	0.0003 (13)	−0.0043 (14)
C18	0.0384 (19)	0.045 (2)	0.0327 (18)	0.0081 (15)	0.0006 (14)	0.0130 (15)

### Geometric parameters (Å, º)

**Table:**

S1—C1	1.642 (3)	C9—H9B	0.9800
S2—C2	1.643 (3)	C9—H9C	0.9800
S3—C1	1.826 (3)	C10—H10A	0.9800
S3—S4	1.9931 (10)	C10—H10B	0.9800
S4—C2	1.828 (3)	C10—H10C	0.9800
N1—C1	1.337 (3)	C11—C12	1.536 (4)
N1—C7	1.474 (3)	C11—H11A	0.9900
N1—C3	1.476 (3)	C11—H11B	0.9900
N2—C2	1.341 (3)	C12—C13	1.516 (4)
N2—C15	1.466 (3)	C12—C14	1.521 (4)
N2—C11	1.469 (3)	C12—H12	1.0000
C3—C4	1.522 (4)	C13—H13A	0.9800
C3—H3A	0.9900	C13—H13B	0.9800
C3—H3B	0.9900	C13—H13C	0.9800
C4—C6	1.510 (4)	C14—H14A	0.9800
C4—C5	1.527 (4)	C14—H14B	0.9800
C4—H4	1.0000	C14—H14C	0.9800
C5—H5A	0.9800	C15—C16	1.532 (4)
C5—H5B	0.9800	C15—H15A	0.9900
C5—H5C	0.9800	C15—H15B	0.9900
C6—H6A	0.9800	C16—C17	1.510 (4)
C6—H6B	0.9800	C16—C18	1.516 (4)
C6—H6C	0.9800	C16—H16	1.0000
C7—C8	1.514 (4)	C17—H17A	0.9800
C7—H7A	0.9900	C17—H17B	0.9800
C7—H7B	0.9900	C17—H17C	0.9800
C8—C10	1.506 (4)	C18—H18A	0.9800
C8—C9	1.520 (4)	C18—H18B	0.9800
C8—H8	1.0000	C18—H18C	0.9800
C9—H9A	0.9800		
			
C1—S3—S4	104.71 (9)	H9B—C9—H9C	109.5
C2—S4—S3	104.22 (9)	C8—C10—H10A	109.5
C1—N1—C7	121.1 (2)	C8—C10—H10B	109.5
C1—N1—C3	125.3 (2)	H10A—C10—H10B	109.5
C7—N1—C3	113.6 (2)	C8—C10—H10C	109.5
C2—N2—C15	119.3 (2)	H10A—C10—H10C	109.5
C2—N2—C11	123.9 (2)	H10B—C10—H10C	109.5
C15—N2—C11	116.6 (2)	N2—C11—C12	114.6 (2)
N1—C1—S1	126.7 (2)	N2—C11—H11A	108.6
N1—C1—S3	111.45 (18)	C12—C11—H11A	108.6
S1—C1—S3	121.80 (15)	N2—C11—H11B	108.6
N2—C2—S2	125.8 (2)	C12—C11—H11B	108.6
N2—C2—S4	112.26 (19)	H11A—C11—H11B	107.6
S2—C2—S4	121.90 (16)	C13—C12—C14	111.6 (2)
N1—C3—C4	113.4 (2)	C13—C12—C11	113.9 (2)
N1—C3—H3A	108.9	C14—C12—C11	107.2 (2)
C4—C3—H3A	108.9	C13—C12—H12	108.0
N1—C3—H3B	108.9	C14—C12—H12	108.0
C4—C3—H3B	108.9	C11—C12—H12	108.0
H3A—C3—H3B	107.7	C12—C13—H13A	109.5
C6—C4—C3	112.5 (2)	C12—C13—H13B	109.5
C6—C4—C5	111.4 (2)	H13A—C13—H13B	109.5
C3—C4—C5	108.8 (2)	C12—C13—H13C	109.5
C6—C4—H4	108.0	H13A—C13—H13C	109.5
C3—C4—H4	108.0	H13B—C13—H13C	109.5
C5—C4—H4	108.0	C12—C14—H14A	109.5
C4—C5—H5A	109.5	C12—C14—H14B	109.5
C4—C5—H5B	109.5	H14A—C14—H14B	109.5
H5A—C5—H5B	109.5	C12—C14—H14C	109.5
C4—C5—H5C	109.5	H14A—C14—H14C	109.5
H5A—C5—H5C	109.5	H14B—C14—H14C	109.5
H5B—C5—H5C	109.5	N2—C15—C16	114.4 (2)
C4—C6—H6A	109.5	N2—C15—H15A	108.7
C4—C6—H6B	109.5	C16—C15—H15A	108.7
H6A—C6—H6B	109.5	N2—C15—H15B	108.7
C4—C6—H6C	109.5	C16—C15—H15B	108.7
H6A—C6—H6C	109.5	H15A—C15—H15B	107.6
H6B—C6—H6C	109.5	C17—C16—C18	111.2 (2)
N1—C7—C8	112.5 (2)	C17—C16—C15	112.8 (2)
N1—C7—H7A	109.1	C18—C16—C15	108.2 (2)
C8—C7—H7A	109.1	C17—C16—H16	108.2
N1—C7—H7B	109.1	C18—C16—H16	108.2
C8—C7—H7B	109.1	C15—C16—H16	108.2
H7A—C7—H7B	107.8	C16—C17—H17A	109.5
C10—C8—C7	113.3 (3)	C16—C17—H17B	109.5
C10—C8—C9	112.2 (3)	H17A—C17—H17B	109.5
C7—C8—C9	109.4 (2)	C16—C17—H17C	109.5
C10—C8—H8	107.2	H17A—C17—H17C	109.5
C7—C8—H8	107.2	H17B—C17—H17C	109.5
C9—C8—H8	107.2	C16—C18—H18A	109.5
C8—C9—H9A	109.5	C16—C18—H18B	109.5
C8—C9—H9B	109.5	H18A—C18—H18B	109.5
H9A—C9—H9B	109.5	C16—C18—H18C	109.5
C8—C9—H9C	109.5	H18A—C18—H18C	109.5
H9A—C9—H9C	109.5	H18B—C18—H18C	109.5

### Hydrogen-bond geometry (Å, º)

**Table:**

*D*—H···*A*	*D*—H	H···*A*	*D*···*A*	*D*—H···*A*
C3—H3*A*···S3	0.99	2.34	2.907 (3)	115
C7—H7*A*···S1	0.99	2.60	3.084 (3)	110
C8—H8···S1^i^	1.00	2.97	3.834 (3)	146
C11—H11*A*···S4	0.99	2.33	2.896 (3)	115
C11—H11*B*···S2^ii^	0.99	2.87	3.810 (3)	160
C16—H16···S2	1.00	2.91	3.473 (3)	117

Symmetry codes: (i) *x*+1, *y*, *z*; (ii) *x*−1, *y*, *z*.
